# Disruption of PML Nuclear Bodies Is Mediated by ORF61 SUMO-Interacting Motifs and Required for Varicella-Zoster Virus Pathogenesis in Skin

**DOI:** 10.1371/journal.ppat.1002157

**Published:** 2011-08-25

**Authors:** Li Wang, Stefan L. Oliver, Marvin Sommer, Jaya Rajamani, Mike Reichelt, Ann M. Arvin

**Affiliations:** Departments of Pediatrics and Microbiology & Immunology, Stanford University School of Medicine, Stanford, California, United States of America; University of Glasgow, United Kingdom

## Abstract

Promyelocytic leukemia protein (PML) has antiviral functions and many viruses encode gene products that disrupt PML nuclear bodies (PML NBs). However, evidence of the relevance of PML NB modification for viral pathogenesis is limited and little is known about viral gene functions required for PML NB disruption in infected cells *in vivo*. Varicella-zoster virus (VZV) is a human alphaherpesvirus that causes cutaneous lesions during primary and recurrent infection. Here we show that VZV disrupts PML NBs in infected cells in human skin xenografts in SCID mice and that the disruption is achieved by open reading frame 61 (ORF61) protein via its SUMO-interacting motifs (SIMs). Three conserved SIMs mediated ORF61 binding to SUMO1 and were required for ORF61 association with and disruption of PML NBs. Mutation of the ORF61 SIMs in the VZV genome showed that these motifs were necessary for PML NB dispersal in VZV-infected cells *in vitro*. *In vivo*, PML NBs were highly abundant, especially in basal layer cells of uninfected skin, whereas their frequency was significantly decreased in VZV-infected cells. In contrast, mutation of the ORF61 SIMs reduced ORF61 association with PML NBs, most PML NBs remained intact and importantly, viral replication in skin was severely impaired. The ORF61 SIM mutant virus failed to cause the typical VZV lesions that penetrate across the basement membrane into the dermis and viral spread in the epidermis was limited. These experiments indicate that VZV pathogenesis in skin depends upon the ORF61-mediated disruption of PML NBs and that the ORF61 SUMO-binding function is necessary for this effect. More broadly, our study elucidates the importance of PML NBs for the innate control of a viral pathogen during infection of differentiated cells within their tissue microenvironment *in vivo* and the requirement for a viral protein with SUMO-binding capacity to counteract this intrinsic barrier.

## Author Summary

PML nuclear bodies (PML NBs) are spherical nuclear structures that are present in most human and animal cells. These bodies contribute to anti-viral defense and therefore many viruses have developed strategies to disrupt them. This interaction has been demonstrated for a number of viruses in cultured cells but little is known about these processes in differentiated cells within human tissues. Varicella-zoster virus (VZV) is a human alphaherpesvirus that causes chicken pox and shingle lesions in skin. Here we show that VZV disrupts PML NBs in epidermal and dermal cells in skin tissues implanted subcutaneously in immunodeficient mice. We found that PML NB dispersal is mediated by VZV ORF61 protein and is required for VZV cell to cell spread and lesion formation in skin. The ability of ORF61 to disrupt PML NBs depends on its capacity to bind to SUMO1 protein, which is conjugated to PML and other proteins within PML NBs. To our knowledge, our study provides the first evidence of PML NB modification through the SUMO-binding function of a viral protein, VZV ORF61, and the importance of this molecular mechanism for virus-induced PML NB disruption in differentiated cells infected within their tissue microenvironment *in vivo*.

## Introduction

Promyelocytic leukemia nuclear bodies (PML NBs), also called nuclear domain 10 (ND10) bodies, are spherical nuclear structures that are present in most mammalian cells [1]. PML protein is the essential major component and recruits other proteins, such as Sp100, Daxx, SUMO1, CBP and p53 [2]. PML NBs are associated with many cellular processes, including transcription, the DNA damage response, apoptosis and oncogenesis [1]. Under normal conditions, the number of PML NBs varies depending on cell type and differentiation status [3]-[5]. PML is also an interferon (IFN)-inducible protein and IFN treatment increases the number and size of PML NBs [6]. PML NBs have been shown to contribute to innate defenses against a broad range of viruses [7]. In turn, many viruses encode products that modify or eliminate PML NBs in cultured cells [7]. However, few studies have investigated the role of PML NBs in viral pathogenesis or mechanisms by which they are modified during viral infection *in vivo* [8], [9].

Varicella-zoster virus (VZV) is the etiologic agent of varicella (chickenpox) and herpes zoster (shingles) and causes characteristic cutaneous lesions in both diseases [10]. VZV is an alphaherpesvirus closely related to herpes simplex virus (HSV) 1 and 2 [10]. PML is known to interfere with HSV early viral gene transcription and it is important for the antiviral effects of IFNs on HSV [11], [12]. PML knock-down and over-expression experiments indicate that PML is also involved in restricting VZV replication [9], [13]. In HSV-infected cells, the ICP0 protein triggers the proteasome-dependent degradation of sumoylated PML and Sp100 through ubiqutin E3 ligase activity mediated by its RING domain [14]-[17]. In contrast to HSV, VZV does not degrade PML protein [9], [13], although it does disrupt PML NBs, causing a reduction of approximately five-fold in PML NB frequencies *in vitro* [9]. Our recent work demonstrated that the PML NBs that persist in VZV infected cells *in vitro* and in skin and dorsal root ganglia (DRG) *in vivo* have the capacity to sequester newly formed nucleocapsids [9]. The entrapment of VZV capsids in these nuclear PML cages depended upon an interaction between PML and the ORF23 capsid protein and acted as an intrinsic antiviral host defense [9].

The VZV ortholog of HSV ICP0 is ORF61 [10]. Like ICP0, ORF61 colocalizes with PML NBs shortly after virus entry and disperses Sp100 NBs in transfected cells if the conserved RING domain is intact [18], [19]. The ORF61 RING domain also exhibits E3 ligase activity *in vitro* [19], [20]. Besides these functions, ORF61 has been shown to act as a transactivator to regulate a number of viral and cellular promoters in transient transfection assays and contributes to the optimal expression of VZV glycoprotein E during virus replication in cultured cells [21]-[23]. ORF61 is essential for VZV replication *in vitro*; deleting ORF61 is not compatible with recovery of infectious virus and truncating ORF61 or severely limiting its expression by mutating promoter elements markedly impairs virus replication [22]-[24].

Although VZV is a highly human-restricted virus, its pathogenesis can be investigated using xenografts of human skin, thymus and DRG in severe combined immunodeficiency (SCID) mice [25], [26]. The evaluation of VZV recombinant viruses with genetic mutations in the SCID mouse model makes it possible to analyze mechanisms of virus-host cell interactions in differentiated cells *in vivo* and to determine whether putative functional motifs in viral proteins or promoters contribute to the capacity of the virus to overcome intrinsic barriers. Targeted mutations in the viral genome that have little or no effect in VZV replication in cultured cells can disrupt functions that are critical for pathogenesis [27]. By evaluating ORF61 promoter mutants in the skin xenograft model, we demonstrated that ORF61 is necessary for VZV skin pathogenesis [23], but the reason for this ORF61 requirement was not defined. In this study, our goal was to investigate the functional elements of ORF61 protein, which are required for interaction with PML NBs in differentiated cells infected *in vivo* and the contribution of this interaction to VZV infection in skin using the xenograft model.

To identify potential ORF61 functional domains, we analyzed the ORF61 sequence and found that it has three putative small ubiquitin-like modifier (SUMO)-interacting motifs (SIMs) in addition to the conserved RING domain. SIMs have been identified in a number of proteins and have a hydrophobic core, consisting of 3-4 aliphatic residues (V/L/I-x-V/L/I-V/L/I or V/L/I-V/L/I-x-V/L/I; ‘x’ means any amino acid), which are typically flanked by a stretch of negatively charged amino acids [28]-[30]. Structural studies indicate that the motif has an extended configuration and is embedded in the groove formed between the α-helix and the β-strand of SUMO [31]. PML protein contains a SIM and the binding through the SIM to sumoylated PML is considered to be the nucleation event for recruitment of other sumoylated and SIM-containing proteins [32]. In support of this model, SIMs in cellular proteins, including Daxx, RNF4 and Sizn1 and in the Kaposi's sarcoma herpesvirus (KSHV) LANA2 protein, are necessary for their association with or modification of PML NBs [33]-[36].

This study was designed to investigate whether the putative SIMs that we identified in ORF61 mediated ORF61 binding to SUMO and if so, whether these SIMs were important for ORF61 association with PML NBs and for the disruption of PML NB *in vitro*. These questions were addressed by mutagenesis of the SIMs in ORF61 plasmid constructs or in ORF61 in the context of the VZV genome. We then examined the significance of the ORF61 SUMO-binding capacity for PML NB association and dispersal in differentiated skin cells using the SCID mouse model. In summary, we have demonstrated that ORF61 has a SIM-mediated SUMO-binding capacity which is necessary for its capacity to target and disrupt PML NBs in differentiated skin cells *in vivo* and that this ORF61 SIM-mediated function is a critical determinant of the pathogenic potential of VZV in skin.

## Results

### ORF61 is a SUMO-binding protein and binds to SUMO1 through conserved SUMO-interacting motifs (SIMs)

**Figure 1:**
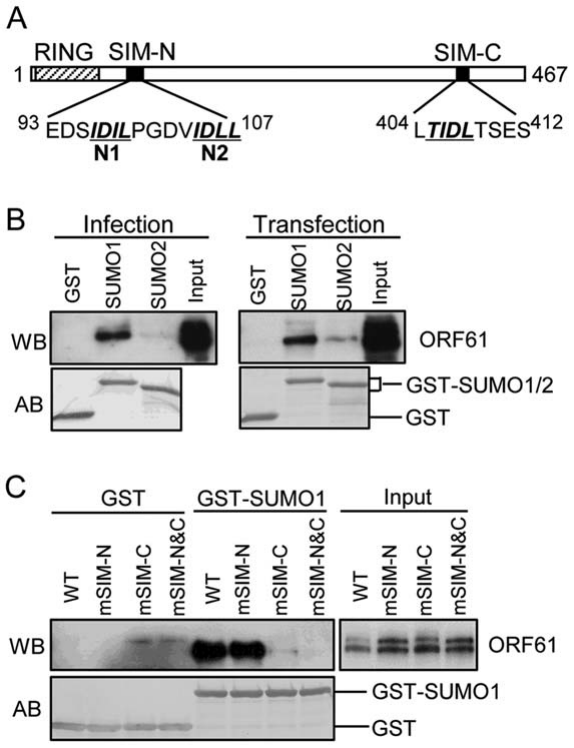
ORF61 binds to SUMO1/2 through conserved SUMO-interacting motifs (SIMs). (A) Schematic representation of the ORF61 protein sequence (amino acids 1-467). The diagonal shaded box indicates the RING domain; filled boxes indicate the three conserved SIMs; the sequences of the SIM hydrophobic core are highlighted by italics and underlined. The numbers in superscript show the amino acid positions of the SIMs in ORF61. (B) Binding of ORF61 (from VZV-infected or ORF61-transfected cell lysates) to SUMO1/2 in GST pull down. Western blot with ORF61 antibody shows the ORF61 proteins bound to SUMO1/2; amido black (AB) staining shows the amount of GST and GST-SUMO1/2 proteins used for binding. (C) Binding of ORF61 SIM mutant proteins from transfected cell lysates to SUMO1 in GST pull down. Western blot shows the amount of ORF61 or SIM mutant proteins bound to SUMO1 and the amount of proteins in the input lysates; amido black (AB) staining shows amount of GST and GST-SUMO1 proteins used for binding.

By sequence analysis, we observed that ORF61 has three putative SIMs; two are located in the N-terminus near the RING domain (designated as SIM-N1 and SIM-N2) and a third is in the C-terminus (designated as SIM-C) (Fig. 1A). Of interest, one or more putative SIMs are also present in the ORF61 orthologs of other alphaherpesviruses (Table 1).

**Table 1:** Prediction of putative SIMs in ORF61 orthologs of other alphaherpesviruses.

Virus	Putative SIMs
HSV-1	^164^IVGV^167^, ^176^IPIV^179^, ^331^VGVV^334^, ^651^VVAL^654^, ^667^IPIL^670^, ^681^VVLV^684^
BHV-1	^173^LPLL^176^, ^252^LLFV^255^, ^489^VIDL^492^
EHV-1	^142^LALV^145^, ^309^IIDL^312^, ^357^IICL^360^, ^435^VVLV^438^
MHV-1	^423^LVGL^426^, ^447^IVDL^450^
FHV-1	^335^LDLT^338^, ^353^VIAL^356^
MDV-1	^147^VALL^150^
PRV	^183^IVEI^186^, ^402^IDLT^405^

HSV-1: herpes simplex 1; BHV-1: bovine herpesvirus 1; EHV-1: equid herpesvirus 1; MHV-1: macropodid herpesvirus 1; FHV-1: felid herpesvirus 1; MDV-1: Marek's disease virus type 1; PRV: pseudorabies virus. The numbers in superscript indicate the amino acid position of each SIM in the protein.

We first investigated whether the ORF61 SIMs had the functional capacity to mediate ORF61 binding to SUMO. In GST pull down assays, ORF61 expressed in VZV-infected cells and in transiently transfected cells bound to GST-SUMO1 and also to GST-SUMO2, although less efficiently; non-specific binding to GST was not detected (Fig. 1B). The ORF61 SIMs were then mutated by alanine substitutions in the ORF61 plasmid, resulting in mSIM-N (both SIM-N1 and SIM-N2 disrupted), mSIM-C (SIM-C disrupted), and mSIM-N&C (all three sites disrupted). When these SIM mutants were evaluated with GST pull down assay, the SUMO1 binding capacity of mSIM-N was similar to ORF61 whereas binding of mSIM-C was reduced markedly and mSIM-N&C did not bind to SUMO1 (Fig. 1C).

### ORF61 SIMs are essential for ORF61 association with PML NBs and ORF61-mediated PML NB dispersal in transfected cells

**Figure 2:**
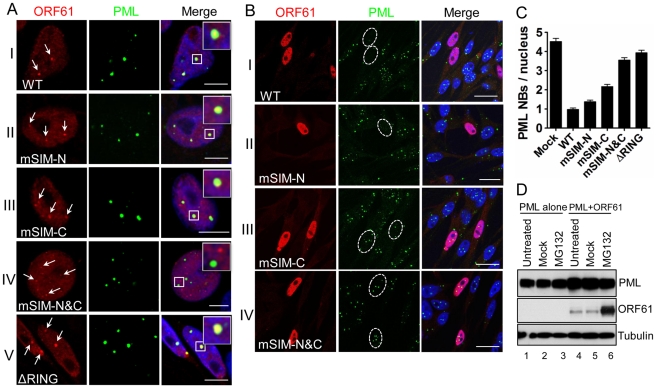
ORF61 SIMs are essential for ORF61 association with PML NBs and PML NB disruption in transfected cells. (A) Confocal microscopy of ORF61-, SIM mutants-, or ▵RING-transfected melanoma cells that were stained for ORF61 (red), PML (green), and nuclei (blue). ORF61-positive puncta in cells expressing low level of ORF61 wild-type and mutant proteins are indicated by white arrows and their associations with PML NBs are shown. The insets are zoom-in images of the white square area. Scale bars: 5 µm. (B) Confocal microscopy of ORF61- or SIM mutant-transfected melanoma cells that were stained for ORF61 (red), PML (green), and nuclei (blue). Nuclei with abundant expression of ORF61 or SIM mutant proteins are outlined by dashed ovals. Scale bars: 20 µm. (C) PML NB frequencies (mean±SEM) in cells expressing abundant ORF61, ▵RING, or SIM mutant proteins (Mock, N = 245; WT, N = 224; mSIM-N, N = 234; mSIM-C, N = 237; mSIM-N&C, N = 238; ΔRING, N = 201). (D) The effect of ORF61 on the PML IV protein level. Melanoma cells transfected with PML IV expressing plasmid alone or together with pcDNA-ORF61 were treated with 10 µM MG132 for 6 h at 24 h post-transfection. Western blots with ORF61, PML, and tubulin antibodies are shown.

Since ORF61 is expressed and colocalizes with PML NBs at very early times in VZV-infected cells [18], we investigated the pattern of ORF61 association with PML NBs in transfected melanoma cells. As expected, distinct ORF61 nuclear puncta were observed in cells with low levels of expression and most colocalized with PML NBs (Fig. 2A, panel I). The effect of the ORF61 SIM mutations on association with PML NBs was then evaluated. The localization of mSIM-N puncta with PML NBs was similar to ORF61 (Fig. 2A, panel II); mSIM-C nuclear puncta were found in only a few cells but also colocalized with PML NBs (Fig. 2A, panel III). In contrast, mSIM-N&C nuclear puncta, also found in only a few cells, did not colocalize with PML NBs (Fig. 2A, panel IV). Examination of an ORF61 mutant that lacked the previously characterized RING/E3 ligase domain [19], [20] showed that this ΔRING mutant formed large bright nuclear puncta, most of which were associated with PML NBs (Fig. 2A, panel V). Thus, in contrast to the ORF61 SIMs, the ORF61 RING domain was not required for PML NB association.

Next, the capacity of ORF61 and SIM mutants to disperse PML NBs was examined. In cells with abundant ORF61 expression, punctate staining was less evident, the protein was distributed more diffusely in the nucleus and the number of PML NBs was decreased significantly compared to cells in the same monolayer that did not express ORF61 (Fig. 2B, panel I). The PML NB frequency in ORF61-positive cells was 0.97±0.08 (N = 224), which was a 4.7-fold reduction (p<0.0001) compared to mock-transfected cells (4.53±0.17, N = 245) (Fig. 2C). PML NBs were also obviously reduced in cells expressing abundant mSIM-N compared to untransfected cells but were better preserved in cells with abundant mSIM-C or mSIM-N&C (Fig. 2B, panel II to IV). The ΔRING mutant had no obvious effect on PML NBs (data not shown), which was consistent with a previous report using Sp100 as a PML NB marker [19]. As determined by quantification of PML NB frequencies in these cells, the capacity of mSIM-N to disperse PML NBs was slightly less than ORF61, the effect of mSIM-C was reduced significantly and mSIM-N&C was completely ineffective (Fig. 2C). The PML NB frequency in mSIM-N&C-expressing cells was comparable to that in cells expressing ORF61 ΔRING and mock-transfected cells (Fig. 2C).

Taken together, these experiments demonstrated that when expressed in the absence of other viral proteins, ORF61 targets and disrupts a majority of PML NBs and that the ORF61 SIMs were necessary and as important as the RING domain for ORF61-mediated dispersal of PML NBs. As a single motif, the C-terminal SIM was the most important for ORF61 SUMO-binding and PML NB dispersal but all three SIMs were required to achieve both the association with PML NBs and their efficient dispersal.

To investigate the effect of ORF61 on PML protein levels, ORF61 was co-expressed with each of six PML isoforms in melanoma cells for 24 h and cells were then treated with the proteasome inhibitor MG132 for 6 h. As shown for PML IV, the PML protein level was unchanged between mock-treated and MG132-treated cells (Fig. 2D, lanes 5 & 6). Similar results were obtained with PML isoforms I-III, V and VI (data not shown). ORF61 accumulated abundantly (Fig. 2D), confirming its rapid turnover [20]. These data suggested that, unlike HSV ICP0, ORF61 does not degrade PML protein despite its conserved RING domain and E3 ligase activity, which is consistent with the persistence of PML protein in VZV-infected cells [9], [13].

### ORF61 SIMs are dispensable for other ORF61 functions

**Figure 3:**
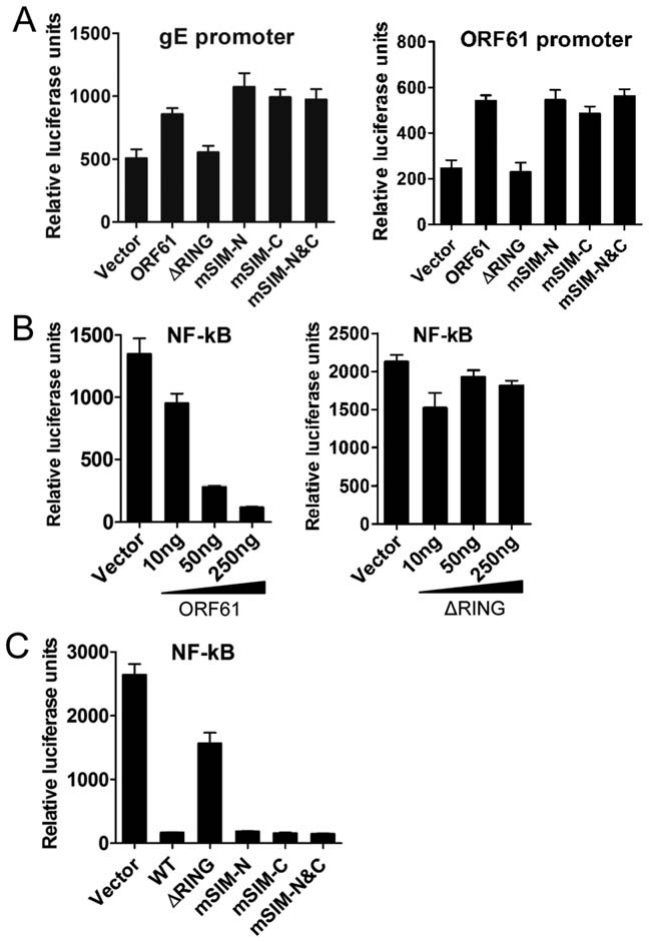
ORF61 SIMs are dispensable for other ORF61 functions. (A) The effect of ORF61 SIM mutants on gE promoter (left panel) and ORF61 promoter (right panel). Luciferase assays were performed on melanoma cells transfected with gE promoter or ORF61 promoter construct and pcDNA constructs expressing ORF61 ΔRING mutant and SIM mutants. (B) NF-κB suppression by ORF61 and ΔRING mutant. Luciferase assays were performed on melanoma cells that were cotransfected with NF-κB reporter plasmid and increasing amounts (10-250 ng) of pcDNA-ORF61 (left panel) or pcDNA-ORF61(ΔRING) (right panel) and then treated with TNF-α. (C) NF-κB suppression by ORF61 SIM mutants. Luciferase assays were performed on melanoma cells that were transfected with NF-κB reporter plasmid and a fixed amount (250 ng) of pcDNA constructs expressing ORF61 SIM mutants and then treated with TNF-α. The graphs show the relative luciferase units (*firefly* luciferase units normalized with *renilla* luciferase units).

We next investigated the potential role of the ORF61 SIMs in other ORF61 functions. ORF61 is known to regulate expression of the essential VZV glycoprotein, gE, through a RING domain-dependent mechanism [21], [37]. However, disrupting the ORF61 SIMs did not alter activation of either the gE or the ORF61 promoters (Fig. 3A). Since VZV inhibits NF-κB activation [38], we also investigated ORF61 regulation of the NF-κB promoter. Of interest, NF-κB activation by TNF-α was suppressed by ORF61 in a dose-dependent manner and again, this function required the RING domain but not the SIMs (Fig. 3B and 3C). Thus, while the RING domain is essential for all of these ORF61 functions, the SIMs are specifically needed for ORF61 association with and dispersal of PML NBs. These data also suggest that the SIM mutations did not result in aberrant folding of ORF61 protein, which would be expected interfere with these other functions.

### ORF61 SIMs are essential for PML NB disruption in VZV-infected cells but are not required for VZV replication *in vitro*

**Figure 4:**
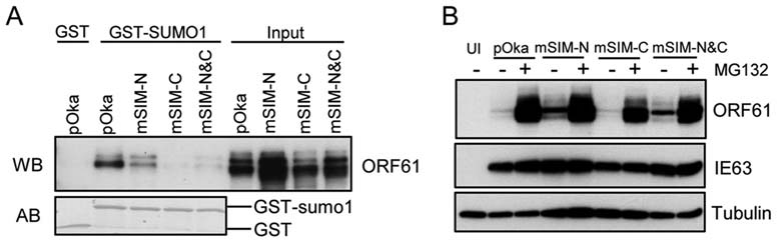
ORF61 SIMs are essential for the SUMO-binding capacity of ORF61 expressed in VZV-infected cells. (A) Binding of ORF61 SIM mutant proteins produced from infected cells to SUMO1 in GST pull down. Western blot with ORF61 antibody shows the ORF61 and SIM mutant proteins bound to SUMO1/2; amido black (AB) staining shows GST and GST-SUMO1/2 levels. (B) The processing of ORF61 and SIM mutant proteins in infected cells. Melanoma cells infected with pOka and ORF61 SIM mutant viruses were mock-treated or treated with MG132. Western blots of ORF61, IE63 (a VZV immediate early protein), and tubulin are shown. IE63 serves as the control for VZV infection. UI indicates uninfected cells.

In order to assess the contributions of the ORF61 SIMs to PML NB association and dispersal in infected cells and to VZV replication, we made three VZV recombinants that contained the same ORF61 SIM mutations as in the ORF61 mutant plasmids. These mutants were designated as pOka-mSIM-N, pOka-mSIM-C and pOka-mSIM-N&C, respectively. Evaluation of the SUMO1-binding capacity of the ORF61 SIM mutant proteins produced in infected cells with the GST pull-down assay showed that interactions of mSIM-C and mSIM-N&C with SUMO1 were almost undetectable whereas mSIM-N binding was diminished only slightly (Fig. 4A), confirming the SIM-mediated binding of ORF61 to SUMO1 as was observed in transfection experiments. Treating cells infected with pOka and ORF61 SIM mutants with MG132 showed that the intracellular processing of the ORF61 SIM mutant proteins was indistinguishable from ORF61 (Fig. 4B), again suggesting that these mutant proteins were not likely to have major structural changes and that their reduced SUMO-binding capacity was attributable to disruption of the functional SIMs.

**Figure 5:**
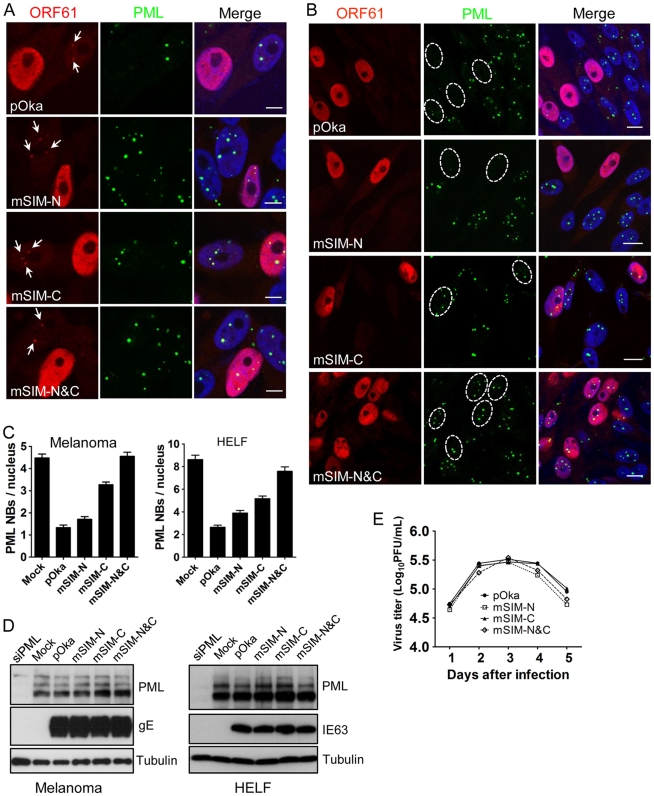
ORF61 SIMs are essential for PML NB disruption in VZV-infected cells but not required for VZV replication *in vitro*. (A) Confocal microscopy of melanoma cells that were infected with pOka and ORF61 SIM mutants for 6 h and stained for ORF61 (red), PML (green), and nuclei (blue). The images show association of puncta (indicated by white arrows) formed by ORF61 and SIM mutant proteins with PML NBs. Scale bars: 5 µm. (B) Confocal microscopy of melanoma cells that were infected with pOka and ORF61 SIM mutants for 24 h and stained for ORF61 (red), PML (green), and nuclei (blue). The images show PML NB dispersal in pOka- or SIM mutant-infected cells. Infected nuclei with abundant expression of ORF61 or SIM mutant proteins are outlined by dashed ovals. Scale bars: 10 µm. (C) PML NB frequencies (mean±SEM) in pOka/ORF61 SIM mutants-infected melanoma cells (Mock, N = 213; pOka, N = 206; mSIM-N, N = 212; mSIM-C, N = 202; mSIM-N&C, N = 232) and HELFs (Mock, N = 227; pOka, N = 221; mSIM-N, N = 235; mSIM-C, N = 225; mSIM-N&C, N = 214). (D) Expression of PML in melanoma cells and HELFs that were infected by pOka or ORF61 SIM mutants. VZV gE or IE63 serve as the control for VZV infection. (E) Replication of pOka and ORF61 SIM mutant viruses in melanoma cells over 5 days. Each point represents mean±SEM of virus titers from three replicates.

When the effect of the SIM mutations on ORF61 association with PML NBs was examined in melanoma cells at 6 h after infection, all three ORF61 SIM mutants formed minute ORF61-positive nuclear puncta, colocalizing with PML NBs (Fig. 5A). After 24 h, PML NBs were reduced substantially in cells infected with pOka-mSIM-N as well as pOka, whereas disruption of PML NBs was considerably less in pOka-mSIM-C- and pOka-mSIM-N&C-infected cells (Fig. 5B). Quantitative analysis showed that cells infected with pOka-mSIM-C retained 2.5-fold more PML NBs compared to pOka-infected cells; the PML NB frequency in cells infected with pOka-mSIM-N&C was equivalent to that in mock-infected cells (Fig. 5C, left panel). Similar patterns were observed in HELFs (Fig. 5C, right panel). These experiments indicate that the capacity of ORF61 to bind to SUMO1 through its SIMs correlates with how efficiently it disrupts PML NBs in VZV-infected cells. The preservation of some colocalization of the mSIM-N&C protein with PML NBs in infected cells, which was not observed under transfection conditions, suggests that another viral factor(s) or a virus-induced factor(s) may also contribute to ORF61 association with PML NBs. Since neither pOka infection nor ORF61 expression alters the levels of endogenous PML protein (9, Fig. 2D), it was expected that PML levels in melanoma cells and HELFs infected with the ORF61 SIM mutants would be unchanged (Fig. 5D).

Despite the compromised effect on PML NB dispersal, the growth kinetics of all three ORF61 SIM mutants was similar to pOka in melanoma cells, as determined by infectious focus assay (Fig. 5E). Yields of ORF61 SIM mutants and pOka were also comparable in HELFs (data not shown). Thus, the dispersal of PML NBs mediated through ORF61 SIMs is not required for VZV replication in permissive cells *in vitro*.

### ORF61 SIMs are required for VZV pathogenesis in skin *in vivo*

**Figure 6:**
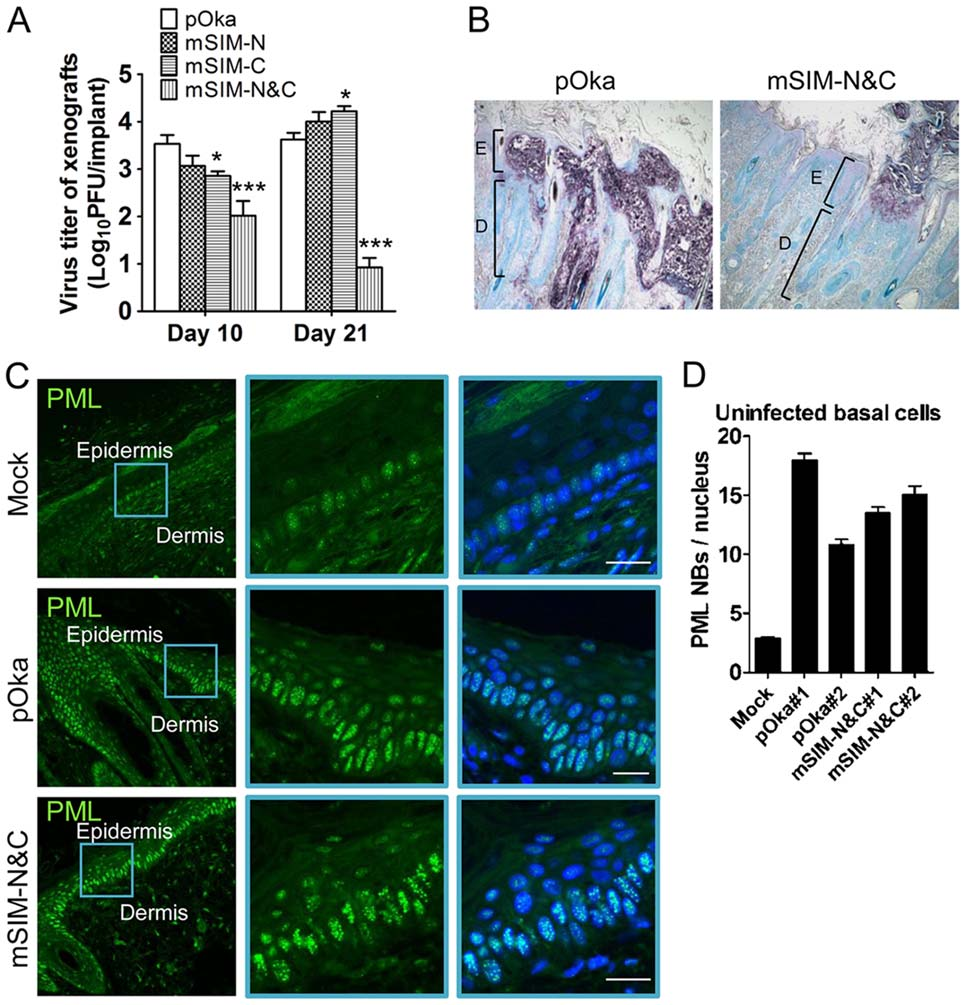
ORF61 SIMs are critical for VZV virulence in skin. (A) Replication of pOka and ORF61 SIM mutant viruses in human skin xenografts in SCID mice (inoculum: ∼8×10^4^ PFU/xenograft). The graph shows the mean titer±SEM of xenografts that yielded viruses at 10 and 21 days post-infection. *, p<0.05; ***, p<0.001; vs pOka (two-way ANOVA). (B) Lesions in skin xenografts infected with pOka- or pOka-mSIM-N&C for 21 days were identified by VZV gE expression. Nuclei were stained with methyl green. Magnification, 5x. Structures of epidermis [E] and dermis [D] are indicated. (C) Confocal microscopy of uninfected cells from mock-infected skin xenografts (upper panels) and pOka- or pOka-mSIM-N&C-infected skin xenografts (middle and lower panels). These skin sections were stained for PML proteins (green) and nuclei (blue). Epidermal and dermal layers are indicated. Middle and right images of each panel are zoom-in of the blue square area from the left image. Scale bars: 20 µm. (D) PML NB frequency (mean±SEM) in uninfected basal cells from each of two skin xenografts infected with either pOka or pOka-mSIM-N&C was quantified and compared with a mock-infected xenograft (number of nuclei examined: Mock, N = 112; pOka#1, N = 99; pOka#2, N = 93; mSIM-N&C#1, N = 109; mSIM-N&C#2, N = 85).

When the ORF61 SIM mutants were evaluated in human skin xenografts in SCID mice, the infectious virus yield of pOka-mSIM-N was similar to pOka, while the replication of pOka-mSIM-C was delayed slightly, with virus yields that were lower than pOka at day 10 (p<0.05) but had increased by day 21 (p<0.05) (Fig. 6A). Infectious virus was recovered from 5 of 6 or all 6 xenografts inoculated with pOka, pOka-mSIM-N or pOka-mSIM-C. In contrast, pOka-mSIM-N&C replication in skin was severely impaired. pOka-mSIM-N&C was recovered from only 4 of 6 inoculated xenografts at day 10 and 3 of 6 at day 21. Titers from the xenografts that yielded pOka-mSIM-N&C were ∼30-fold lower than pOka at day 10 (p<0.001) and ∼500-fold lower at day 21 (p<0.001) (Fig. 6A).

Analysis of skin tissue sections showed that pOka formed the usual large necrotic VZV lesions that penetrate across the basal cell layer into the dermis whereas pOka-mSIM-N&C lesions were very small and restricted to the epidermis (Fig. 6B). It is important to note that PML NBs were prominent in skin cells in mock-infected xenografts, with the highest frequency being found in cells of the basal layer (Fig. 6C, upper panels). However, many more PML NBs were present in the uninfected cells present within skin xenografts that were infected with pOka or pOka-mSIM-N&C, compared to these mock-infected tissues (Fig. 6C, middle and lower panels and Fig. S1). Quantification of two skin xenografts that were infected with pOka and two infected with pOka-mSIM-N&C showed 3.8-6.6 fold increase in PML NB frequency in uninfected cells respectively, compared with the mock-infected xenograft (Fig. 6D); the PML NB frequencies did not differ significantly between xenografts inoculated with pOka or pOka-mSIM-N&C (Fig. 6D). Since skin cells dramatically up-regulate IFN production in response to VZV infection *in vivo* and PML is IFN-inducible [6], [39], our findings suggest that the innate IFN response increases PML NB numbers in dermal and epidermal cells, reinforcing a pre-existing barrier to VZV spread.

### ORF61 SIMs are required for ORF61 association with PML NBs and their dispersal in skin cells

**Figure 7:**
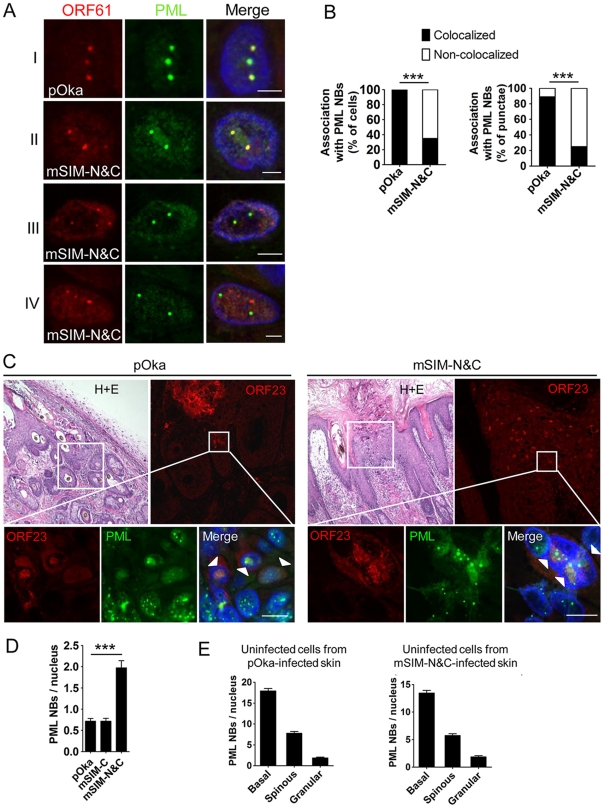
ORF61 SIMs are required for ORF61 association with and dispersal of PML NBs in skin cells *in vivo*. (A) Confocal microscopy of pOka- or pOka-mSIM-N&C-infected skin cells that were stained for ORF61 (red), PML (green), and nuclei (blue). The images show association of ORF61-positive puncta with PML NBs in newly infected cells. Scale bars: 2.5 µm. (B) Quantitation of ORF61/PML NB association in pOka- or pOka-mSIM-N&C-infected cells that had ORF61-positive puncta. The left panel shows percentage of cells that show/did not show ORF61 colocalization with PML NBs (pOka, N = 47; mSIM-N&C, N = 46; ***, p<0.0001 by Chi-square). The right panel shows percentage of ORF61-positive puncta colocalized/not colocalized with PML NBs (pOka, N = 167; mSIM-N&C, N = 202; ***, p<0.0001 by Chi-square). (C) Confocal microscopy of pOka- or pOka-mSIM-N&C-infected skin cells that were stained for ORF23 (red), PML (green), and nuclei (blue). For each panel (left and right), the upper left image shows skin structures of a parallel section by hematoxylin and eosin (H+E) staining; the upper right image shows epidermal cells from the white square area on the upper left image (the infected cells were identified by ORF23 expression); the lower images show PML NBs in ORF23-positive cells and neighboring uninfected cells from the white square area on the upper right image. White arrows indicate cells with abundant nuclear ORF23 protein. Scale bars: 10 µm. (D) PML NB frequencies (mean±SEM) in pOka- or ORF61 SIM mutants-infected skin cells (pOka, N = 153; mSIM-C, N = 143; mSIM-N&C, N = 108; ***, p<0.0001 by *t*-test). (E) PML NB frequencies (mean±SEM) in uninfected skin cells from pOka- or pOka-mSIM-N&C-infected skin xenografts. Cells from the three major epidermal layers were grouped. Uninfected cells from pOka-infected skin: basal layer cells (N = 99), spinous layer cells (N = 79), granular layer cells (N = 45). Uninfected cells from pOka-mSIM-N&C-infected skin: basal layer cells (N = 109), spinous layer cells (N = 124), granular layer cells (N = 39).

The small epidermal lesions and the failure of pOka-mSIM-N&C to create lesions that extend across the basal layer suggested that VZV spread from cell to cell in skin might depend on ORF61 SIM-mediated PML NB disruption. To investigate this possibility, we examined the pattern of ORF61/PML NB association and the PML NB frequencies in skin cells infected with pOka and the ORF61 SIM mutants. ORF61 nuclear puncta that colocalized with PML NBs were evident in the newly infected cells located at the margins of pOka skin lesions (Fig. 7A, panel I). pOka-mSIM-N&C also formed distinct ORF61-positive nuclear puncta but in contrast to pOka, the pattern of their association with PML NBs was heterogenous in individual cells (Fig. 7A, panel II-IV). Skin cells that had mSIM-N&C puncta could be categorized into three groups: those in which either all or some puncta colocalized with PML NBs (Fig. 7A, panel II and III) and those in which none colocalized with PML NBs (Fig. 7A, panel IV). When these associations were quantitated in the skin cell nuclei that had ORF61 or mSIM-N&C puncta, colocalization of ORF61 with PML NBs was observed in 100% of pOka-infected cells (N = 47) whereas only 35% of pOka-mSIM-N&C-infected cells (N = 46) showed any colocalization of ORF61 with PML NBs (p<0.0001) (Fig. 7B, left panel). Analyzing these data based on total numbers of ORF61 positive puncta in infected cell nuclei showed that 89% of pOka ORF61 puncta (N = 167) were associated with PML NBs compared to only 25% of mSIM-N&C puncta (N = 202) (p<0.0001) (Fig. 7B, right panel).

Next, the frequency of PML NBs in skin cells infected with pOka and the ORF61 SIM mutants was assessed. ORF23, a VZV nucleocapsid protein, was used as a marker for infected cells as abundant ORF23 protein indicates a later stage of VZV infection [10]. Only infected cells with intact nuclear membranes were analyzed to exclude those in which VZV had induced necrosis. PML NBs in pOka-infected cells were reduced compared with uninfected cells in the same section (Fig. 7C, left panels), while the frequency of PML NBs in pOka-mSIM-N&C-infected cells was similar to uninfected cells (Fig. 7C, right panels). Quantitative analysis showed that more PML NBs (2.8-fold higher) were preserved in pOka-mSIM-N&C-infected cells compared to pOka-infected cells (pOka, 0.71±0.07, N = 153; pOka-mSIM-N&C, 1.97±0.17, N = 108; p<0.0001) (Fig. 7D). The PML NB frequency in pOka-mSIM-C-infected cells (N = 143) was equivalent to pOka-infected cells (Fig. 7D). Both the detection of PML expression and the frequency of PML NBs were similar in the uninfected cells within xenografts infected either with pOka or pOka-mSIM-N&C (Fig. 6C and 7E). This finding makes it unlikely that the difference in PML NB frequency in skin cells infected by these two viruses reflects a variation of PML NB frequencies between individual xenografts.

Taken together, these results demonstrate that ORF61 SIMs determine the capacity of VZV to target and disrupt PML NBs in skin cells *in vivo* and indicate that VZV spread in skin depends on this function.

### Growth of ORF61 SIM mutant viruses in IFN-treated cultured cells

**Figure 8:**
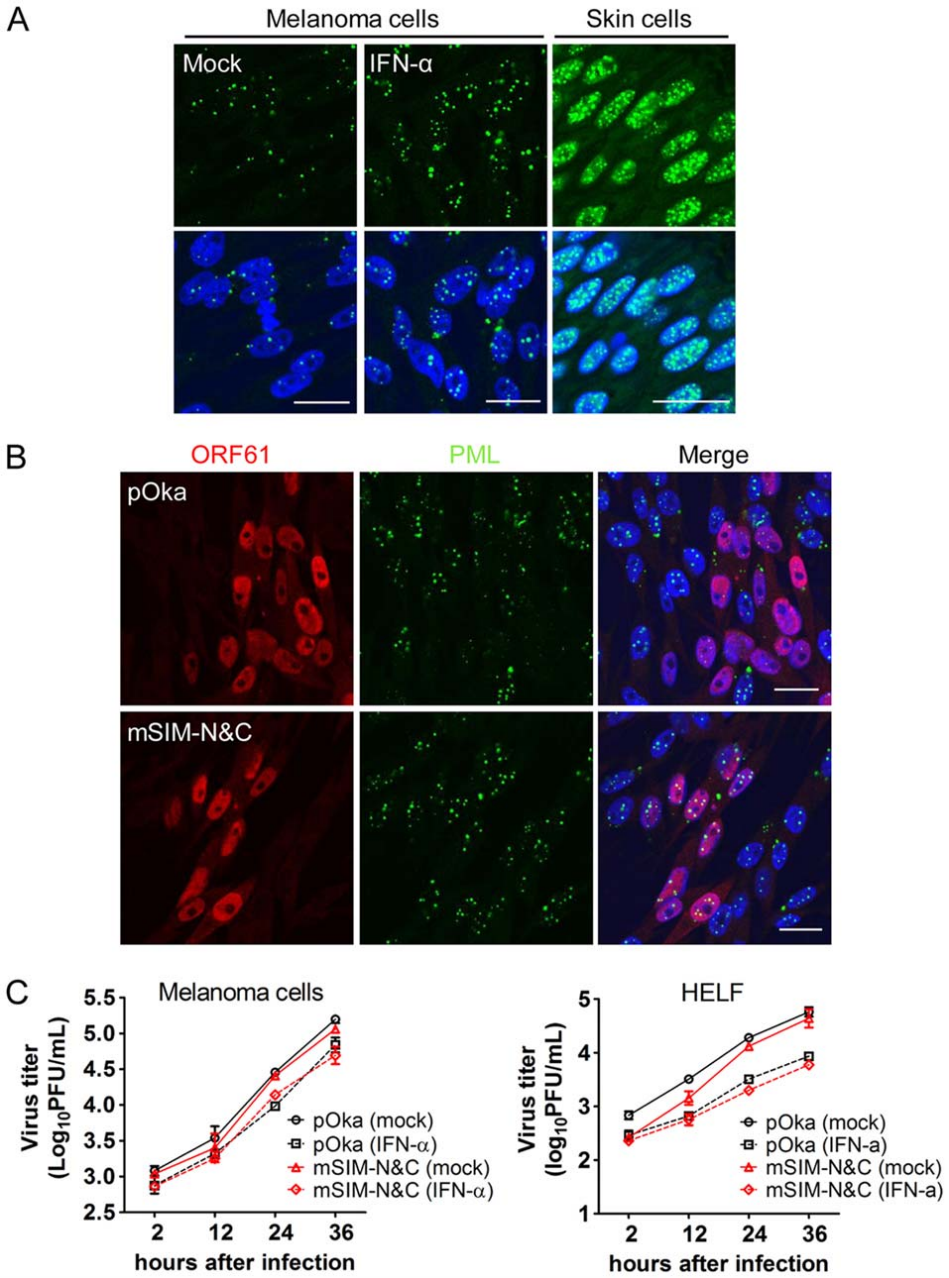
ORF61 SIM-dependent disruption of PML NBs is not required for VZV replication in cultured cells treated with IFN-α. (A) Confocal microscopy of mock- or IFN-α-treated melanoma cells that were stained for PML (green) and nuclei (blue) (left and middle panels). Right panel shows PML NBs (green) in uninfected cells from pOka-infected skin xenografts. Nuclei were stained with Hoechst (blue). Scale bars: 20 µm. (B) Confocal microscopy of melanoma cells that were treated with IFN-α and then infected with pOka or pOka-mSIM-N&C. Cells were stained for ORF61 (red), PML (green), and nuclei (blue). The images show PML NBs in cells expressing abundant ORF61 and SIM mutant proteins. Scale bars: 20 µm. (C) Replication of pOka and pOka-mSIM-N&C in melanoma cells (left panel) and HELF cells (right panel) treated with IFN-α. Each point represents mean±SEM of virus titers from three replicates.

Since these experiments in skin xenografts showed that pOka-mSIM-N&C had a growth deficiency *in vivo* which was not detectable *in vitro* and IFN-α, which induces PML, is present in VZV-infected xenografts *in vivo* but is not produced by VZV-infected cells *in vitro* [39], [40], we evaluated the replication of pOka and pOka-mSIM-N&C in cultured cells treated with IFN-α. The number of PML NBs was increased by IFN-α treatment and as expected, the PML NBs were disrupted by pOka but not by pOka-mSIM-N&C (Fig. 8A and 8B). Nevertheless, IFN-α treatment had a comparable effect on reducing pOka and pOka-mSIM-N&C titers in melanoma cells and HELFs (Fig. 8C). However, even with IFN treatment, PML NB frequencies in melanoma cells remained significantly lower than frequencies observed in skin cells *in vivo* (Fig. 8A). That pOka-mSIM-N&C was not differentially inhibited in IFN-α treated cells when compared to pOka *in vitro*, in contrast to the severely impaired growth of pOka-mSIM-N&C in skin supports the significance of the very high frequencies of PML NBs that are present in dermal and epidermal cells for limiting the spread of VZV *in vivo*.

### Growth of ORF61 SIM mutant viruses in non-proliferating cells *in vitro*

**Figure 9:**
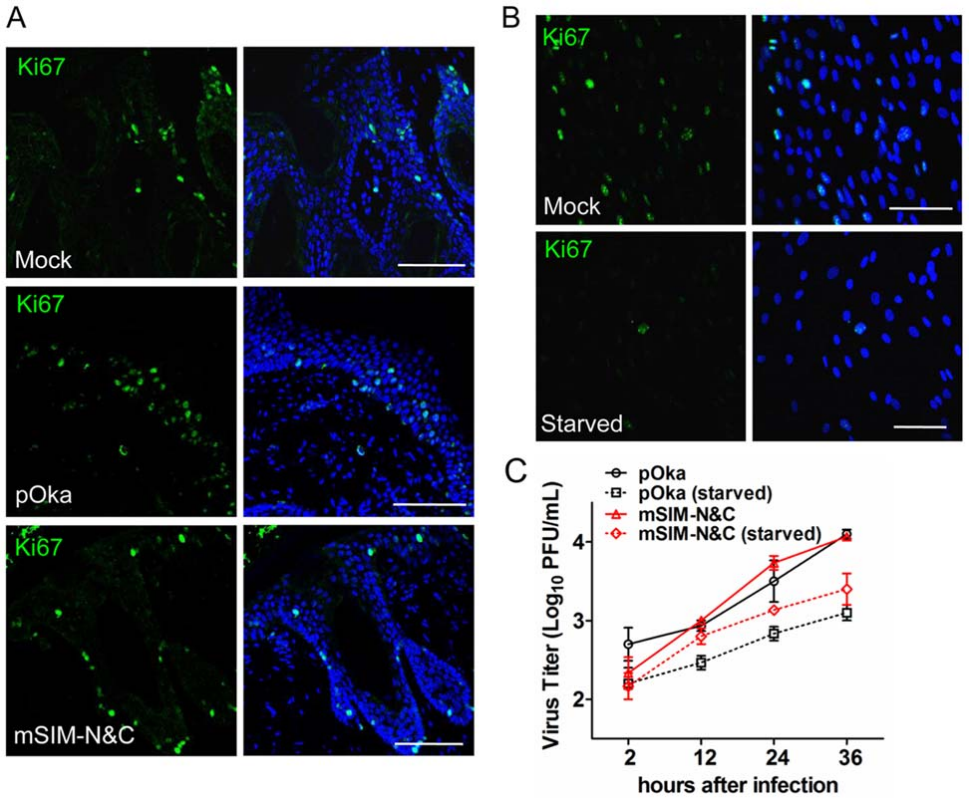
ORF61 SIMs are not required for VZV replication in non-proliferating cells *in vitro*. (A) Confocal microscopy of mock-, pOka-, or pOka-mSIM-N&C-infected skin xenografts that were stained for the proliferation marker Ki67 (green) and nuclei (blue). Scale bars: 100 µm. (B) Confocal microscopy of normal and serum-starved HELFs that were stained for the proliferation marker Ki67 (green) and nuclei (blue). Scale bars: 100 µm. (C) Replication of pOka and pOka-mSIM-N&C in normal and serum-starved HELFs. Each point represents mean±SEM of virus titers from three replicates for each virus under normal and serum-starved conditions.

Since VZV, like other viruses, replicates more efficiently in proliferating cells, another reason for the defective growth of the ORF61 SIM mutants in skin compared to cultured cells might be fewer proliferating cells *in vivo*. Based on the expression of Ki67, a proliferation marker [41], most HELFs were proliferating under standard cell culture conditions whereas only a few skin cells from mock- or VZV-infected xenografts were Ki67-positive (Fig. 9A and 9B). No significant difference in Ki67 expression was observed between uninfected cells in pOka- or pOka-mSIM-N&C-infected xenografts (Fig. 9A). To further investigate whether the ORF61 SIMs confer an advantage for VZV replication in non-proliferating cells, the growth kinetics of pOka and pOka-mSIM-N&C were compared in serum-starved HELFs. The percentage of Ki67-positive HELFs was reduced to a minimal level by serum starvation (Fig. 9B). However, titers of pOka-mSIM-N&C in serum-starved HELFs were indistinguishable from pOka titers (Fig. 9C). These results suggested that the difference in the importance of ORF61 SIMs for VZV replication in skin compared with cultured cells did not reflect an advantage for VZV replication in non-proliferating cells that depends upon the ORF61 SIMs.

## Discussion

This study provides the first direct evidence of the importance of PML NB disruption for the efficient replication of a viral pathogen in differentiated human cells located within their usual tissue microenvironment *in vivo* and of the requirement for the SUMO-binding capacity of a viral protein, VZV ORF61, to counteract the innate antiviral control mediated by PML NBs. This function is critical in VZV skin infection because the persistence of the virus in the human population depends upon its capacity to produce cutaneous lesions that contain high concentrations of infectious virus particles for transferring to susceptible individuals [10]. Notably, we found that PML NBs were highly abundant in human skin cells, especially in the basal cell boundary between the epidermis and the dermis. In the absence of functional ORF61 SIMs, these PML NBs were preserved and VZV infection was severely impaired. Thus, our experiments indicate that VZV pathogenesis in skin requires ORF61 association with and dispersal of PML NBs and that this modification of host cell nuclear structures depends upon ORF61 SIMs. We suggest that PML NB-mediated control of VZV replication must be counteracted by ORF61 in order to produce the characteristic cutaneous lesions of varicella and herpes zoster and that ORF61 SUMO-binding capacity is necessary for this essential phase of the VZV life cycle in the human host.

More generally, the finding that ORF61 has functional SIMs that modulate PML NBs provides new evidence that non-covalent SIM-SUMO interactions can alter the structure and dynamics of PML NBs [32]-[36]. Importantly, the evaluation of VZV ORF61 SIM-deficient mutants in skin xenografts shows that this SIM-dependent effect on PML NBs occurs in differentiated cells within tissues *in vivo*. To our knowledge, ORF61 is the first example showing that a viral RING finger protein has functional SIMs, like those that are present in cellular RING finger proteins, such as RNF4, which is involved in PML degradation [34]. Of interest, the less conserved SIM-C motif ‘TIDL’, which has also been identified in other SUMO-binding proteins [42], appeared to be more critical than the other two highly conserved ORF61 SIMs. One possible explanation is the access of SIM-N1 and SIM-N2 sites to SUMO1 might be hindered since they are in a proline-rich region and proline may interrupt the β-strand conformation required for the interaction. Our analysis of ORF61 SIM-C provides additional evidence that the less conserved ‘TIDL’ motif can be a functional SIM and demonstrates its role in a viral SUMO-binding protein.

In previous work, we found that VZV infection in skin causes a dramatic upregulation of IFN-α in uninfected cells surrounding VZV lesions and that this response is critical for controlling infection, as shown by enhanced VZV replication and extensive skin lesion formation when the IFN pathway is blocked [39]. We now report that PML NBs are also increased substantially from an already high baseline when skin is infected with VZV. Since PML expression is regulated by IFN, these observations are further evidence of the significant role of IFN-mediated innate immunity in skin [39]. Our data indicate that PML NBs are an important mechanism by which IFN control of VZV infection is achieved *in vivo* and that ORF61 SIM-mediated targeting and dispersal of PML NBs is necessary to counteract this innate response during VZV pathogenesis. These observations also help to explain why antiviral immunity was defective in a PML knockout mouse model [8].

The finding that the growth of pOka-mSIM-N&C was severely defective in skin xenografts, whereas it was indistinguishable from pOka in cultured cells, illustrates that the functional significance of the ORF61 SIMs could only be demonstrated when assessed in the context of VZV replication in differentiated host cells *in vivo*. The very high frequencies of PML NBs in differentiated skin cells *in vivo* suggests that the abundance of PML NBs determines whether the ORF61 SIM-mediated effect on PML NBs is necessary for VZV to achieve efficient replication. PML NB frequencies could not be enhanced to these levels in cultured cells even with IFN treatment *in vitro*. Since PML NBs are also numerous in the nuclei of both neurons and satellite cells [9], it will be of interest to investigate the contribution of ORF61 SIM-dependent PML NB disruption in VZV neuropathogenesis. The need to define functional requirements for disrupting PML NBs *in vivo* was also evident from the fact that mutating SIM-C alone was sufficient to substantially affect ORF61 dispersal of PML NBs in cultured cells whereas all three SIMs were necessary for PML NB disruption in skin. Another difference between cell culture and skin cells *in vivo* was that the mutant ORF61 lacking all SIMs retained some capacity to associate with PML NBs in cultured cells but the association was reduced significantly in skin cells. These observations suggest another VZV protein(s) or VZV-activated cellular factor(s) may bind to ORF61 and facilitate ORF61 targeting of PML NBs *in vitro* but has a limited role *in vivo*. We speculate that the differential availability or expression of these factors in skin cells compared to cultured cells might have contributed to the differential requirement of the three ORF61 SIMs to VZV replication *in vivo* and *in vitro*. These questions warrant further investigation, particularly in the different types of human cells that are targeted during VZV pathogenesis.

In HSV-infected cells, the ICP0 RING domain acts as an ubiquitin E3 ligase and triggers proteasome-dependent PML degradation and PML NB disruption [14], [15]. Our experiments confirmed that the RING domain was also necessary for ORF61-induced PML NB dispersal but we found that it was dispensable for ORF61 association with PML NBs. In contrast, the ORF61 SIMs were required for both PML NB association and disruption, indicating that PML NB dispersal by ORF61 is a two-step process: the ORF61 SIMs first recognize sumoylated PML protein in PML NBs and the RING domain is needed to execute dispersal. As was consistent with the persistent PML protein expression in VZV-infected cells [9], [13], ORF61 expression as a single protein caused PML NB disruption but PML protein was not degraded; therefore, we speculate that the ORF61 RING domain has functions other than E3 ligase that are necessary for its contribution to PML NB disruption. For example, since RING domains mediate protein-protein interactions [43], the RING domain in ORF61 may form a complex with other RING finger proteins in the nucleoplasm and thereby dislodge ORF61 SIM-bound PML proteins from PML NBs. However, during other cellular events in which ORF61 is involved, it is quite possible that the ORF61 RING domain acts as an E3 ligase and mediates its substrate degradation, since ORF61 RING domain is known to exhibit E3 ligase activity in vitro [19], [20]. It is known that all alphaherpesviruses encode RING finger proteins related to ICP0 and ORF61 and most of them have been shown to target PML NB components [44]. Of interest, our sequence analyses indicate the presence of conserved SIM(s) in these RING finger proteins. Taken together, we propose that the interaction of these SIM-containing viral proteins with sumoylated PML is conserved and essential for alphaherpesviruses to target PML NB structures for dispersal whereas the RING domain in the orthologs has variable functions, and these functions determine whether, or the extent to which, PML protein is degraded. Our recent experiments in VZV-infected skin cells and neurons showed that PML protein that persists can form a novel class of PML nuclear cages with the capacity to sequester newly synthesized capsids and constitute an intrinsic anti-VZV defense [9]. Viruses like HSV have the capability to completely overcome the PML-mediated intrinsic barrier as they not only disrupt PML NBs but also efficiently degrade PML protein; therefore these viruses may have an advantage against the IFN defense that is triggered and amplified within infected tissues. We speculate that this difference in the effect on PML protein, resulting from the different functional capacities of ICP0 and ORF61, may help to explain why HSV reactivates much more frequently than VZV in the naturally infected host [45]. Of note, the gammaherpesvirus KSHV-encoded LANA2 protein disperses PML NBs by a SIM-dependent process in cultured cells although it does not have a RING domain [36]. We propose that SIM-dependent disruption of the architecture of PML NBs might be a conserved mechanism among herpesviruses, independent of the fate of PML protein.

As noted, many viruses encode gene products that have been shown to modify PML NBs in cultured cells [7]. However, to define the specific importance of PML NB modulation for viral replication and pathogenesis, viral mutants that are disabled only in this function are needed. HSV ICP0 RING domain mutant viruses do not disrupt PML NBs but the RING domain is also essential for other ICP0 functions, including viral gene expression, innate immune evasion and virion formation [46]-[48]. Therefore, it is difficult to attribute the replication defect of the HSV ICP0 RING domain mutant to a single function. Experiments with the ORF61 SIM mutants avoided these concerns because the RING domain was intact and other functions of ORF61, including viral gene regulation and NF-κB suppression and ORF61 protein stability were preserved. These ORF61 functions, such as NF-κB regulation, are also likely to be important contributions of this multi-functional protein to VZV pathogenesis and warrant further investigation [23]. Nevertheless, since ORF61 SIMs might target many more sumoylated proteins in PML NBs or other nuclear compartments, other ORF61 SIM-mediated interactions could contribute to VZV replication *in vitro* and to pathogenesis *in vivo* in addition to their role in PML NB disruption.

In summary, we have demonstrated that VZV ORF61 interacts with SUMO1 via three conserved SIMs. The interaction was essential for the association of ORF61 with PML NBs and ORF61-mediated PML NB disruption in infected skin cells *in vivo*. VZV replication and spread in skin tissues *in vivo* depended on this ORF61 SIM-mediated function. Our data support the conclusion that PML NBs provide an intrinsic host defense against VZV infection in skin, which is an essential stage in VZV pathogenesis during primary and recurrent infection of the human host and we have elucidated the ORF61 SIM-dependent mechanism that is used by VZV to counteract these antiviral nuclear structures.

## Materials and Methods

### Ethics statement

The human fetal tissues for SCID xenograft studies were obtained from Advanced Bioscience Resources, Inc. (Alameda, CA) in accordance with state and federal regulations for tissue acquisition for biomedical research, in accordance with FDA 21 CFR Part 1271 GTP (Good Tissue Practices), UAGA and NOTA. Human melanoma cells (a tumor cell line) and primary human embryonic lung fibroblasts (HELF) were derived and used as described previously [23]. Animal protocols complied with the Animal Welfare Act and were approved by the Stanford University Administrative Panel on Laboratory Animal Care.

### Cells

Human melanoma cells (a tumor cell line) and primary human embryonic lung fibroblasts (HELF) were derived and used as described previously [23]. The PML knock-down melanoma cell line (siPML) was a gift from Prof. Saul Silverstein, University of Columbia and was used as the negative control in PML western blot experiments.

### Chemicals and reagents

Proteasome inhibitor MG132 (Calbiochem) was diluted in DMSO and used at 10 µM; DMSO was used for mock treatment. Human tumor necrosis factor α (TNF-α) (Biovision) was used at 20 ng/mL for activation of the NF-κB pathway. Human IFN-α Hu-IFN-α2b (PBL InterferonSource) was used at 1000 international units (IU) per mL for upregulation of PML in melanoma cells and HELFs.

### Antibodies

Rabbit polyclonal antibodies against ORF61 and IE63 were kindly provided by Prof. Paul Kinchington, University of Pittsburgh; rabbit polyclonal anti-ORF23 was generated by rabbit immunization with purified protein [49]. PML antibodies were mouse monoclonal anti-PML (PG-M3) and rabbit polyclonal anti-PML (Santa Cruz Biotech). Other antibodies included mouse monoclonals against SUMO1 (Zymed), VZV gE (Chemicon), and α-Tubulin (Sigma) and rabbit polyclonal against Ki67 (Abcam).

### Plasmids

The ORF61 expression plasmid pcDNA-ORF61 was constructed by cloning full-length ORF61 coding sequence into pcDNA3.1(+). ORF61 DNA was amplified by PCR with forward primer (ORF61-F) 5′-GATCAAGCTTATGGATACCATATTAGCGGG-3′ containing a HindIII site and reverse primer (ORF61-R) 5′-GCGAATTCCTAGGACTTCTTCATCTTGT-3′ containing an EcoRI site. The PCR product was digested with HindIII and EcoRI and ligated to HindIII&EcoRI-digested pcDNA3.1(+) to generate pcDNA-ORF61. The ORF61(ΔRING) fragment, from which amino acids 1-60 were deleted, was amplified by PCR with the forward primer (ΔRING-F) 5′-GATCAAGCTTATGGTGCAATCCATCCTGCATAAG-3′ containing the HindIII site and the reverse primer (ORF61-R), and ligated to HindIII/EcoRI-digested pcDNA3.1(+) to generate pcDNA-ORF61(ΔRING). pcDNA constructs expressing ORF61 SIM mutants (pcDNA-mSIM) were constructed by substituting the four hydrophobic core residues of SIMs with alanines in the context of pcDNA-ORF61 (SIM-N1, IDIL to AAAA; SIM-N2, IDLL to AAAA; SIM-C, TIDL to AAAA). Primers used for SIM mutagenesis are:

SIM-N1: 5′-CATTTGAAGATTCCGCTGCCGCTGCACCGGGAGATG-3′ and 5′-CATCTCCCGGTGCAGCGGCAGCGGAATCTTCAAATG-3′; SIM-N2: 5′-CCGGGAGATGTCGCAGCTGCTGCGCCACCAAGCCCA-3′ and 5′-TGGGCTTGGTGGCGCAGCAGCTGCGACATCTCCCGG-3′; SIM-C: 5′-CGATGCTTAGCAGCAGCCGCGACATCTGAGTCTGA-3′ and 5′-TCAGACTCAGATGTCGCGGCTGCTGCTAAGCATCG-3′

PML mammalian expression plasmids were kind gifts from Prof. Peter Hemmerich, Leibnitz-Institute of Age Research, Germany and Prof. Annie Sittler, Universite Pierre et Marie Curie, France. The NF-κB reporter plasmid was gift from Prof. Dingxiang Liu in Institute of Molecular and Cell Biology, Singapore. The gE promoter luciferase construct was as described [50]. All constructs were confirmed by nucleotide sequencing (Elim Biopharm, Inc., Hayward, CA).

### GST pull down

ORF61-transfected cells or VZV-infected cells grown on 10 cm dishes were lysed in 100 ul high salt buffer (20 mM HEPES [pH 7.2], 450 mM NaCl, 1.5 mM MgCl_2_, 0.5% NP-40, 20% Glycerol) supplemented with EDTA-free protease inhibitor cocktail (Roche). Insoluble proteins were removed by centrifugation. The supernantant was combined with 400 ul NaCl-free buffer (20 mM HEPES [pH 7.2], 1.5 mM MgCl_2_, 0.5% NP-40, 20% glycerol) and centrifuged again before GST pull down. GST or GST-SUMO1/2 proteins (5 µg) (Boston Biochem) were added to the pre-cleared lysate and rotated at 4°C for 2 h. Glutathione Sepharose beads (GE Healthcare) were added subsequently and incubated at 4°C for 2 h. Beads were washed 4 times with the binding buffer (20 mM HEPES [pH 7.2], 90 mM NaCl, 1.5 mM MgCl_2_, 0.5% NP-40, 20% glycerol) and eluted with SDS sample buffer. The ORF61 protein that bound to the beads was analyzed by Western blot using the ORF61 antibody and the GST and GST-SUMO1/2 proteins that bound to the beads was analyzed by amido black staining.

### Cosmid mutagenesis and generation of ORF61 SIM mutant viruses

Recombinant viruses were generated using cosmids derived from pOka [51]. The entire pOka genome is covered by four overlapping cosmids designated Fsp73 (pOka nucleotides [nt] 1 to 33128), Spe14 (pOka nt 21795 to 61868), Pme2 (pOka nt 53755 to 96035), and Spe23 (pOka nt 94055 to 125124). The ORF61 coding region is located in the unique long region in the cosmid Spe23 (pOka nt 103045-104445). A 4.8 kb PstI-PmlI fragment containing full length ORF61 gene was subcloned into pCR4-TOPO (Invitrogen, Inc.) to make pCR4-(PstI/PmlI), in which ORF61 SIM mutations were generated using two round PCR method. The PstI-PmlI fragments containing ORF61 SIM mutations were cloned into pLit(ORF59-65) [23]. Mutant Spe23 cosmids were made by ligating the NheI-AvrII fragment from pLit(ORF59-65) to Spe23 digested with NheI/AvrII.

Recombinant viruses, designated pOka-mSIM-N, pOka-mSIM-C, and pOka-mSIM-N&C, were isolated by transfection of melanoma cells with the mutated Spe23 cosmid and the other three intact cosmids, Fsp73, Spe14, and Pme2. Genomic DNA was extracted from virus-infected melanoma cells or HELFs with DNAzol reagent (Invitrogen, Carlsbad, CA). A PCR fragment covering the mutated region was amplified from the genomic DNA using *Taq* polymerase (Invitrogen) and sequenced (Elim Biopharm, Inc., Hayward, CA) to confirm mutations.

### Virus growth kinetics and infectious focus assay

Melanoma cells (10^6^/well) were seeded in a 6-well plate, infected with 1×10^3^ PFU/well at day 0, and cultured for 5 days. On each day, cells from one well were trypsinized, centrifuged, and resuspended in 1 mL of culture medium. The infected cells were serially diluted 10-fold, and 0.1 mL was added to melanoma cells in 24-well plates in triplicate. Cells were fixed in 4% paraformaldehyde and stained with polyclonal anti-VZV human immune serum and secondary anti-human biotin (Vector Lab, Burlingame, CA). The staining was developed with the Fast Red substrate (Sigma). Statistical analysis of growth kinetics was done by the Student's *t* test.

### Infection of skin xenografts in SCIDhu mice

Skin xenografts were made in homozygous CB-17^scid/scid^ mice, using human fetal tissue supplied by Advanced Bioscience Resources (ABR, Alameda, CA) according to federal and state regulations; the methods used to engraft and infect the skin xenografts were as described previously (25). Animal use was in accordance with the Animal Welfare Act and approved by the Stanford University Administrative Panel on Laboratory Animal Care. pOka and ORF61 SIM mutant viruses were passed three times in primary HELF and titered before inoculation. Skin xenografts were harvested at day 10 and 21 and titers were determined by infectious focus assay. DNA was extracted from skin tissues with proteinase K and phenol chloroform (Invitrogen, Carlsbad, CA). PCR and sequencing were performed to confirm the expected mutations.

### Immunofluorescence and PML NB quantification

Cells seeded on 12 mm glass cover slips were fixed with 4% paraformaldehyde for 15 min and permeablized with 0.5% Triton-X100 for 10 min. For dual staining of ORF61 and PML proteins, cells were incubated with ORF61 rabbit polyclonal antibody (1∶200 dilution) and PML mouse monoclonal antibody PG-M3 (1∶50 dilution) in blocking buffer (PBS with 5% fetal bovine serum) at room temperature (RT) for 1 h, followed by incubation with Alexflour 488 donkey anti-mouse immunoglobulin (Invitrogen) and Texas Red-conjugated donkey anti-rabbit immunoglobulin (Jackson ImmunoResearch) for 30 min. Cell nuclei were stained with 2 µg/mL Hoechst33342 for 10 min after secondary antibody incubation. All images were obtained with a SP2 Leica confocal microscope. Images were taken in a fixed setting with the 63x objective in PML NB number quantification experiments, and with the 100x objective to quantify the association between ORF61 puncta and PML NBs in infected skin cells.

For skin experiments, sections (5 µm) were made from formalin-fixed, paraffin-embedded skin tissues. After deparaffinization and rehydration, sections were treated with citrate-based antigen unmasking solution (Vector labs) and stained with specific antibodies (anti-PML [PG-M3], 1∶10; anti-ORF61, 1∶25; anti-ORF23, 1∶100). Skin sections were examined for cell proliferation using Ki67 antibody (Abcam).

### Immunohistochemistry

Skin sections were stained with VZV gE antibody (Chemicon, 1∶2000) and IHC immunoperoxidase secondary detection system (Chemicon). Staining was developed with Vector VIP substrate kit and methyl green counterstain (Vector labs).

### IFN sensitivity assay

Melanoma cells or HELFs were seeded at 5×10^5^ cells/well in 6-well plates. Cells in each well were mock-treated or treated with 1000 IU of Hu-IFN-α2b (PBL InterferonSource) for 24 h prior to inoculation. Cells were inoculated with 500 PFU/well of either pOka or pOka-mSIM-N&C. The media was aspirated at 2 h post-infection to remove the inoculum and replaced with media with or without 1000 IU of IFN-α2b. Virus titers from 2 h to 36 h post-inoculation were determined by infectious focus assay on melanoma cells.

### Serum starvation assay

HELFs were seeded at 5×10^5^ cells/well in 6-well plates and at 2×10^5^ cells/well in 2-well chamber slides. Cells were starved in serum-free culture medium for 48 hours and then inoculated with 50 PFU/cm^2^ of pOka or mSIM-N&C. At 2 hours post infection, the inoculum was removed and the medium was replaced with normal medium or serum-free medium. Virus titers in cells recovered from the 6-well plates at intervals of 2 h to 36 h post-inoculation were determined by infectious focus assay on melanoma cells. Cells on chamber slides were fixed with paraformaldehyde and stained with VZV gE (Chemicon) and Ki67 (Abcam) antibodies.

### Transfection and infection of cultured cells

All transfections were performed with Lipofectamine 2000 (Invitrogen, Carlsbad, CA) following the manufacturer's instructions. The transfected cells for immunofluorescence were fixed at 24 h post-transfection. To prepare heavily infected cells for Western blot analysis of PML proteins, melanoma cells and HELFs were inoculated with pOka or ORF61 SIM mutants at a ratio of 1 infected cell:100 uninfected cells. At 24 h post-inoculation, each cell monolayer was resuspended by trypsinization and re-plated on the same dish and left for another 24 h. The percentage of infected cells was examined by immunofluorescence with ORF23 antibody and was >90% at the time of harvest. To prepare infected cells for immunofluorescence microscopy, cells growing on glass coverslips were inoculated with pOka or ORF61 SIM mutants at a ratio of 1 infected cell: 1000 uninfected cells and infected for 24 h.

### Western blot

Transfected or infected cells growing on 6-well plates were lysed in high salt buffer as described above. Proteins were separated on SDS-PAGE gels and transferred to polyvinylidene difluoride (PVDF) membrane (Millipore, Bedford, MA) with a semidry transfer cell (Bio-Rad). The membranes were blocked for 1 h in 5% non-fat milk in PBST (1x PBS plus 0.1% Tween-20), incubated with primary antibody at RT for 2 h, washed three times with PBST, incubated with horseradish peroxidase-conjugated rabbit or mouse immunoglobulin (Amersham) at RT for 1 h, and washed three times with PBST. Proteins were detected using the enhanced chemiluminescence plus detection system (Amersham).

### Luciferase assay

Melanoma cells were seeded in 24-well plates one day before transfection. Three independent transfections were preformed for each experiment. In the gE and ORF61 promoter assay, 900 ng of the promoter construct was transfected to melanoma cells with 100 ng of pcDNA-ORF61 or pcDNA-mSIM or empty vector for 24 h. In NF-κB experiments, 500 ng of NF-κB reporter plasmid was cotransfected with 250 ng of pcDNA-ORF61 or pcDNA-mSIM or empty vector; at 24 h post-transfection, cells were treated with 20 ng/mL TNF-α for 6 h before cell lysis and luciferase assay. In the NF-κB dose-dependent experiment, increasing amounts of pcDNA-ORF61 or pcDNA-ORF61(ΔRING) (10 ng, 50 ng, and 250 ng) were used and the total DNA was brought to 850 ng with empty vector. In all transfections, 0.07 ng of the plasmid pRL-TK(–) in which TK promoter has been removed was included to normalize the transfection efficiency. Luciferase assays were performed using the dual luciferase kit (Promega) according to the manufacturer's recommendations.

### Accession numbers

VZV ORF61: NP_040183; VZV ORF23: AAY57709.1; VZV IE63: Q77NN7; VZV gE: Q9J3M8.

## Supporting Information

Figure S1 **PML expression in uninfected and infected cells from pOka- or pOka-mSIM-N&C-infected skin xenografts.** Confocal microscopy of pOka- or pOka-mSIM-N&C-infected skin xenografts that were stained for ORF23 (red), PML (green), and nuclei (blue).
(TIF)
