# Supplementary figures and images for: Disruption of PML Nuclear Bodies Is Mediated by ORF61 SUMO-Interacting Motifs and Required for Varicella-Zoster Virus Pathogenesis in Skin
Source: PLoS Pathog. 2011 Aug 25;7(8):e1002157. doi: 10.1371/journal.ppat.1002157 (PMC3161977; doi:10.1371/journal.ppat.1002157)

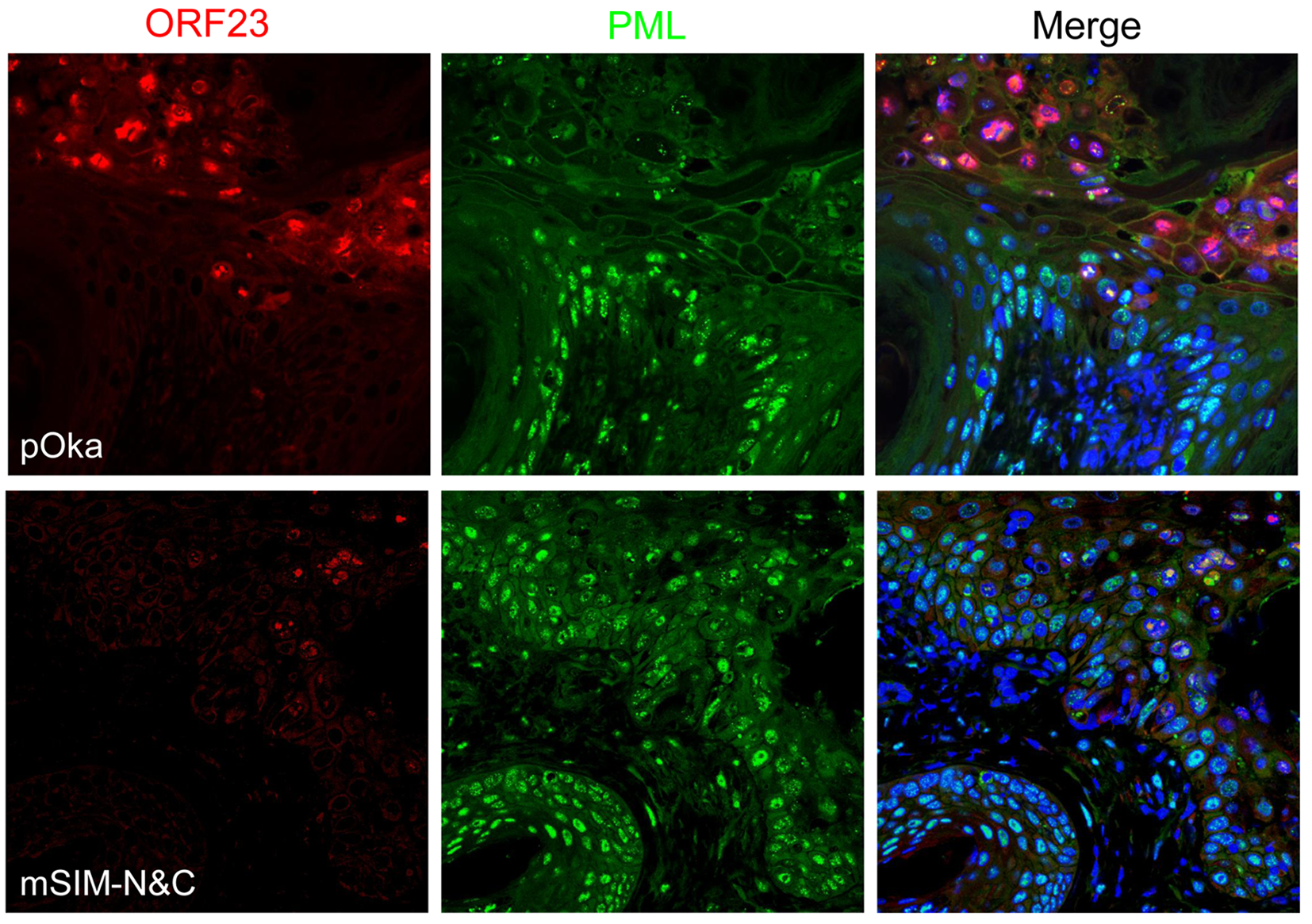

Supplement: Figure S1 — PML expression in uninfected and infected cells from pOka- or pOka-mSIM-N&C-infected skin xenografts. Confocal microscopy of pOka- or pOka-mSIM-N&C-infected skin xenografts that were stained for ORF23 (red), PML (green), and nuclei (blue). (TIF) [file ppat.1002157.s001.tif]
